# Supplementary material for: Global Transcriptomic Profile Analysis of Genes Involved in Lignin Biosynthesis and Accumulation Induced by Boron Deficiency in Poplar Roots
Source: Biomolecules. 2019 Apr 19;9(4):156. doi: 10.3390/biom9040156 (PMC6523340; doi:10.3390/biom9040156)
Supplement: Supplementary file 1 [file biomolecules-09-00156-s001.zip › supplememt files/Fig.s1.doc]

**Figure S1.** Classification of raw reads of two libraries: CK (control, 3-C-1) and BD (boron deficiency, 3-B-18).
