# Supplementary material for: Global Transcriptomic Profile Analysis of Genes Involved in Lignin Biosynthesis and Accumulation Induced by Boron Deficiency in Poplar Roots
Source: Biomolecules. 2019 Apr 19;9(4):156. doi: 10.3390/biom9040156 (PMC6523340; doi:10.3390/biom9040156)
Supplement: Supplementary file 1 [file biomolecules-09-00156-s001.zip › supplememt files/Table s1.doc]

**Table S1** Primers used in qRT-PCR.

| Gene Model | Symbol | Forward primer | Reverse primer | Description |
| --- | --- | --- | --- | --- |
| Potri.001G451100 | *COMT25* | GAGGCACACCTCTTTGCCAT | GGGCCAGCTTTAGCCATGAT | caffeic acid 3-O-methyltransferase |
| Potri.005G117500 | *F5H1* | CCAGGGGTGCCTGATTTCAA | ACAGCCAAATCAAGGGCGTA | ferulate-5-hydroxylase |
| Potri.010G183500 | *LAC* | CACGGTACTACATGGCAGCA | GGCAGCACAAGGGGATGATT | laccase |
| Potri.007G067600 | *MYB192* | TGGGCATGAAAACTGGAGGG | CCACGCTTAACGTCAGGTCT | Myb-related protein |
| Potri.012G127700 | *MYB199* | GCAAGATTGCCGGGAAGAAC | TTGAGAGGCTCGTGCGTAAC | Myb-related protein |
| Potri.009G095800 | *CAD1* | ACCCTCTCGAGCCTTACCTT | TCCCAAGCATAACCATAGGCG | cinnamyl-alcohol dehydrogenase |
| Potri.009G062800 | *CAD5* | TACCACGATGGAACCACCAC | TGCAGCGGCATCTAGAGGTA | cinnamyl-alcohol dehydrogenase |
| Potri.009G076300 | *CCR9* | ACTTGAAGGAGCAGACACGC | GGAGAGGCCAGGTGAAAGAC | cinnamoyl-CoA reductase |
| Potri.002G004500 | *CCR11* | CACGTTGCTTGCCCAATTCC | ACGGAGCAAGCATTCAGTACA | cinnamoyl-CoA reductase |
| Potri.015G003100 | *COMT1* | GGTGCTCTCTTGTCCCCATC | GGAGTAACTAGCCAGAAGGCG | caffeic acid 3-O-methyltransferase |
| Potri.012G006400 | *COMT2* | GAGGCACACCTCTTTGCCAT | GGGCCAGCTTTAGCCATGAT | caffeic acid 3-O-methyltransferase |
| Potri.019G010400^a^ | *ACT2* | ACC CTC CAA TCC AGA CAC TG | TTG CTG ACC GTA TGA GCA AG | similar to actin - garden pea |

a. From Regier N, Streb S, Cocozza C, Schaub M, Cherubini P, Zeeman SC, Frey B. 2009. Drought tolerance of two black poplar (*Populus nigra* L.) clones: contribution of carbohydrates and oxidative stress defence. Plant Cell Environ 32:1724-36.
