# Supplementary material for: Global Transcriptomic Profile Analysis of Genes Involved in Lignin Biosynthesis and Accumulation Induced by Boron Deficiency in Poplar Roots
Source: Biomolecules. 2019 Apr 19;9(4):156. doi: 10.3390/biom9040156 (PMC6523340; doi:10.3390/biom9040156)
Supplement: Supplementary file 1 [file biomolecules-09-00156-s001.zip › supplememt files/Table s2.doc]

**Table S2** Major characteristic of two libraries.

| Summary | Clean Reads | |
| --- | --- | --- |
|  | Control (3-C-1) | BD (3-C-18) |
| Perfect match | 4653757 (40.07%) | 432638 (35.60%) |
| ≤2 bp mismatch | 2965716 (25.53%) | 3252110 (26.76%) |
| Unique match | 313859 (27.02%) | 3090080 (25.43%) |
| Map to gene |  | |
| Total mapped reads | 7619473 (65.60%) | 7578494 (62.36%) |
| Total unmapped reads | 3994868 (34.40%) | 4573636 (37.64%) |
| Multi-position match | 4480875 (38.58%) | 4484414 (36.94%) |
| Map to genome |  |  |
| Total mapped reads | 9106567 (78.41%) | 9474465 (77.97%) |
| Total unmapped reads | 2507774 (21.59%) | 2677665 (22.03%) |
| Multi-position match | 879403 (7.57%) | 955142 (7.86%) |
| Total reads | 11614341 | 12152130 |
